# A Protocol for the Multi-Omic Integration of Cervical Microbiota and Urine Metabolomics to Understand Human Papillomavirus (HPV)-Driven Dysbiosis

**DOI:** 10.3390/biomedicines8040081

**Published:** 2020-04-08

**Authors:** Nataliya Chorna, Filipa Godoy-Vitorino

**Affiliations:** 1 Department of Biochemistry, UPR School of Medicine, 00921 San Juan, Puerto Rico; nataliya.chorna@upr.edu; 2 Department of Microbiology & Medical Zoology, UPR School of Medicine, 00921 San Juan, Puerto Rico

**Keywords:** multi-omics, cervical epithelial microenvironment, HPV, 16S rRNA, metabolomics

## Abstract

The multi-omic integration of microbiota data with metabolomics has gained popularity. This protocol is based on a human multi-omics study, integrating cervicovaginal microbiota, HPV status and neoplasia, with urinary metabolites. Indeed, to understand the biology of the infections and to develop adequate interventions for cervical cancer prevention, studies are needed to characterize in detail the cervical microbiota and understand the systemic metabolome. This article is a detailed protocol for the multi-omic integration of cervical microbiota and urine metabolome to shed light on the systemic effects of cervical dysbioses associated with Human Papillomavirus (HPV) infections. This methods article suggests detailed sample collection and laboratory processes of metabolomics, DNA extraction for microbiota, HPV typing, and the bioinformatic analyses of the data, both to characterize the metabolome, the microbiota, and joint multi-omic analyses, useful for the development of new point-of-care diagnostic tests based on these approaches.

## 1. Introduction

A growing body of evidence, especially over the last twelve years, suggests that the composition and function of the microbiota in different human body habitats play vital roles in human development and immunity [1,2,3]. The vagina and cervix have the lowest biodiversity, with a colonized epithelium dominated by Lactobacilli [4], thus being an interface that plays a protective role between the host and the environment. The cervix serves a pivotal role as a gatekeeper to protect the upper genital tract from microbial invasion and subsequent pathology. Recent studies have indicated that changes of the cervicovaginal microbiome [5,6,7], such as bacterial vaginosis [8,9], cervical inflammation [10,11] and vaginal pH [12,13], play a role in the susceptibility to cervical Human Papillomavirus (HPV) infection and the development of cervical intraepithelial neoplasia due to the profound shifts in the relative abundances of protective bacteria [10]. Although cervical cancer is preventable mainly by vaccination, detection, and treatment of lesions, it remains an important public health problem, being the fourth and the second most common cancer in women globally and in developing countries, respectively (1). In Latin America and the Caribbean, it remains the number one cause of mortality due to malignant neoplasms among 20- to 40-year-old women (2), three times higher in Latin America than in the United States (US) (3). According to guidelines established by Healthy People [2], women that have had a Pap test in three subsequent years need to be at ~90%, however, in some areas of the world such as Latin America, this does not happen, with women often lost to follow-up even before treatment occurs.

Guidelines for genital care include coming to clinics for a Pap smear for HPV typing and cervical cytology, and for those who meet the guidelines, a return for a second visit to receive colposcopy and biopsy. These are invasive and expensive methods, and the approaches explained in this article are innovative and have the potential to be useful in advancing the field. A persistent infection with high-risk human papillomavirus (hr-HPV) types is a common, but not sufficient to cause cervical cancer [14,15]. High risk (hr) HPV infection often requires lifestyle factors for cancer development, such as smoking or oral contraceptive use [14], however, the reasons for hr HPV promotion and persistence are not completely understood. A gap in our understanding of HPV infection and carcinogenesis resides in the insufficient understanding of the true microbial community structure, preventing our ability to control the microbiota for therapeutic purposes. As widely known, the cervicovaginal microbiome also plays a role in disease pathogenesis in combination with HPV, namely genital dysbiosis, a modifiable risk factor for cervical disease [3,4,5,6]. A comparison of the vaginal microbiome in women of different ethnicities within the U.S. mainland indicates different relative abundances of the four main groups of *Lactobacillus* and anaerobic bacteria [4]. Different studies have described that infections with HPV are associated with an increase in *Atopobium vaginae* and *G. vaginallis* [5,11]. Other Latino populations, like Mexicans, have shown *Sneathia spp*. as a cervical biomarker for neoplasia [7]. Not only is bacterial biota associated with HPV, but studies have also found an important role of fungal signatures such as that of *Malassezia* for hr HPV infections and an increase in Sporidiobolaceae and *Sacharomyces* in atypical squamous cells of undetermined significance (ASCUS) lesions [5,16]. Thus, non-*Lactobacillus* dominant communities, with a concomitant increase in pH and related to high-grade dysplasia, perturb amino acid and nucleotide metabolisms and drive inflammation and HPV infections. The combination of the microbiome with metabolome can become even more informative than classical HPV typing. Recently, microbial inventories via sequencing of 16S rRNA genes, have been complemented with metabolomics, adding a layer of information regarding the metabolic activities of the microbiome that helps elucidate the etiology of the microbiome-mediated disease [17,18]. Metabolites produced by commensal microorganisms may affect host metabolic processes, with both pathogenic and protective consequences. Multi-kingdom colonization (bacteria, Archaea, fungi, and viruses) associated with HPV infections in the vagina and cervix can produce metabolites which translocate through the host’s barriers, that could influence systemic health effects in women at risk of developing cervical cancer. Indeed, recent findings suggest that cervical microbial metabolic changes may influence systemic health effects in women at risk of developing cervical cancer [18]. Recent data has been emerging on the usefulness of coupling microbiota inventories with systemic metabolomics in biofluids such as urine (that can be self-collected), thus easy to obtain and minimally invasive. Indeed, biomarkers for cervical cancer may be exfoliated as debris in urine, a kind of liquid biopsy—which could facilitate diagnostics [1]. It is, therefore, important to develop protocols to help integrate information between the microbiome, viral pathogenesis, and immunity affecting cancer progression. Additional to microbiota analyses, one could develop shotgun metagenomic methods to understand the full diversity more accurately, including Archaea and viral composition affecting the dysbiotic or disease status. Indeed, shotgun approaches are useful methods that can non-specifically detect HPVs and other highly divergent viruses (specifically DNA viruses), using rolling circle amplification (RCA) [19], or even using meta-total RNA sequencing (MeTRS) methods based on shotgun sequencing of total RNA, which could reveal the extent of RNA viruses [20] (even the novel coronavirus—SARS-CoV-2), which could lead to new methodologies to reveal the impact of the new virus on the microbiome and the immune response responsible for carcinogenesis. It is crucial to develop more microbiome/metabolome studies to understand cancer progression. The early detection and treatment of genital dysbiosis may facilitate better management of the disease [12]. As such, new quick tests that could monitor genital health based on the use of vaginal secretions and even urine could be useful. Dysbioses of the genital microbiome result in high pH due to the decrease in fermenting *Lactobacillus*, and create a genital microbiome/metabolome fingerprint, which in the future could be detected with different applications [21,22]. These metabolomic profiles require advanced diagnostics and point-of-care applications for which big data from microbiome and metabolome are needed. Here, we provide a unique and detailed guide for the development of multi-omic integration studies of the bacterial microbiota and urine metabolomics to understand possible biological associations with the host’s urinary metabolites. This article discloses detailed materials, methods, sample processing, and data analyses. The coupling of two omic methods is a useful approach to understand the association of the human microbiome with health status and risk of disease severity, and for the development of new point-of-care testing technologies.

## 2. Experimental Section

### 2.1. Materials for Microbiota Production

#### 2.1.1. Patient Recruitment and Sampling

1. With the approved IRB protocol, explain the informed consent verbally to the patient.
2. Deliver the questionnaire.
3. Gather the data collection form—recording the patient health record, sample ID, weight and height, age, and check exclusion criteria such as those suggested in the Manual of Procedures of the Human Microbiome [23]: (1) taken antibiotics in the last month (30 days); (2) have a history of regular urinary incontinence; (3) Treatment for or suspicion of, ever having had toxic shock syndrome; (4) have candidiasis; (5) urinary tract infections; (5) active STD: (6) vaginal irritation at the time of screening and (7) a total hysterectomy (or other criteria specific for each given project).
4. Deliver the educational material (flyer) and the copy of the signed consent.
5. Give the Sterile 4oz Specimen Cups, and ask the patient to collect the urine.
6. During the oB/Gyn visit, during the pelvic examination, labia are spread for introitus visualization, and the specimen is collected by placing one swab at the vagina, posterior to the hymenal ring/tissue. The swab should be rotated along the lumen with a circular motion. Swabs will then be placed in the 1.5 mL Eppendorf.
7. For the cervical samples (posterior fornix), a Pederson speculum is inserted for access and visualization of the cervix. The sterile swab will be placed in the posterior fornix and rotated along the lumen with a circular motion and swabs immediately placed in the sterile tubes.
8. All vaginal and cervical swab samples will be stored at ultra-low temperatures (−80 °C) until DNA extractions.

#### 2.1.2. Collection of Cervical Swabs for Microbiota and HPV Typing

1. Cotton tipped swab applicator with semi-flexible polystyrene handle
2. 1.5mL Eppendorf tubes
3. Zip-Lock bags

### 2.2. Genomic DNA Extraction for Microbiota and HPV Typing

An example of genomic DNA extraction with this protocol is by using a QIAGEN Powersoil kit for low biomass swab samples, according to Human Microbiome standards.

**Figure 1:**
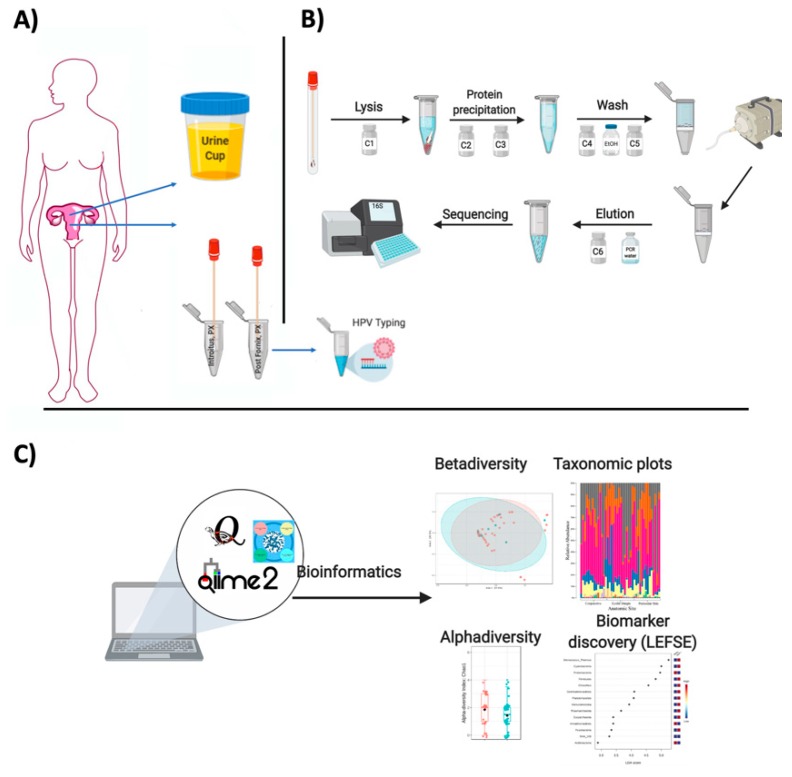
Summary of sample acquisition and analyses. (**A**) shows sample collection in the Ob/Gyn, (**B**) summarizes DNA extraction and (**C**) summarizes data analyses platforms and types of analyses used in microbiota studies.

1. The first step is an alkaline lysis. Solution C1 contains Sodium Dodecyl Sulfate (SDS)—an anionic detergent that breaks down fatty acids and lipids of cell membranes to aid in cell lysis. Should it form a precipitate (cold temperature), just warm the solution in a water bath (up to 60 °C). The solution can be used while it is still warm.
2. Vortex Power Bead tubes with the swabs briefly.
3. Add 60 μL of C1 to the power bead tube with the swab and vortex briefly.
4. Vortex horizontally at maximum speed for 10 min (physical cell disruption).
5. Centrifuge for 30 s at 13,000 rpm.
6. Transfer all supernatant to a clean 2ml collection tube (~750 μL volume).
7. In the Second step (solution C2), what occurs is the precipitation of non-DNA organic and inorganic material, including cell debris and proteins. Given that cervical swabs are low biomass samples, this step will be joined with inhibitor removal (solution C3).
8. Add 100 μL of C2 and 100 μL of C3. Vortex for 5 s.
9. Incubate at −20 °C for 5 min.
10. Centrifuge at room temperature for 1 min at 13,000 rpm.
11. Avoiding the pellet, transfer up to, but no more than 750 μL of supernatant to a clean 2 mL tube.
12. The next step with solution 4 (rich in salt), is aimed at binding the DNA to spin filter.
13. Add 1200 μL of C4 (previously shaken) to the former supernatant and vortex for 5 s.
14. Place the spin filters in the vacuum manifold and load approximately 675 μL onto a spin filter. Turn on the vacuum until all the supernatant flows through. Add an additional 675 μL of the supernatant to the spin filter and vacuum.
15. Load the remaining of the supernatant and repeat it. A total of three loads for each sample processed are required.
16. The next step includes washing the DNA bound to the membrane and remove contaminants.
17. Add 650 μL of cold 100% ethanol (−20 °C) to all the tubes and turn on the vacuum.
18. Add 500 μL of solution C5 to all tubes and turn on the vacuum. Carefully place the spin filter in a clean 2ml collection tube. Avoid splashing any Solution C5 onto the spin filter. It is critical to remove all traces of Solution C5 as it can interfere with many downstream DNA applications.
19. The final step is DNA elution from the membrane using sterile solution C6 (10 mM Tris) or sterile PCR water.
20. Add 60–100 μL of C6 (55 °C) to the center of the white filter membrane and let sit for 5 min. Volume range depends on how diluted you wish the sample to be, If low biomass, lower volumes of C6 are suggested.
21. Centrifuge for 1 min at room temperature at 13,000 rpm.
22. Discard the spin filter and store DNA at −20 °C (Figure 1).

#### 2.2.1. Human Papilloma Virus Genotyping

We suggest the use of a highly sensitive short-polymerase chain reaction-fragment assay (Labo Biomedical Products, Rijswijk, The Netherlands, licensed Innogenetics technology). This assay has been used for epidemiological and vaccination studies because it has high analytical sensitivity [24,25] and other cervicovaginal microbiota related studies [5,26]. It starts with a PCR step, followed by a reverse hybridization protocol.

1. Thaw the DNA previously extracted from clinical samples, mix by vortexing and spin down the tubes to collect all liquid at the bottom of the tube.
2. The assay uses SPF10 primers to amplify a 65-bp fragment of the L1 open reading frame of HPV genotypes, followed by a reverse-hybridization step. The 65-bp PCR fragment assay amplifies the following common mucosal HPV genotypes: 6, 11, 16, 18, 31, 33, 34, 35, 39, 40, 42, 43, 44, 45, 51, 52, 53, 54, 56, 58, 59, 66, 68/73, 70, and 74. This step uses Boxes A and B.
3. Prepare the master mix for the PCR. At first use of the PCR master mix (vial A1), it is recommended to mix the contents of the vial on a Vortex mixer and aliquot the contents in ready-to-use portions of 40 μL. This further reduces the risk of contamination. After aliquoting, store at −20 °C
4. Prepare the tubes/96-well plates (depending on the number of samples) considering the number of the samples to process and include negative and positive controls provided in the kit.
5. Mark each PCR reaction vial/PCR plate.
6. Spin down the liquid in the tubes with the processed specimen for 15 s at 14,000 rpm in a minicentrifuge.
7. Add (one by one) 10 μL of the supernatant of the processed sample to the appropriate vials with PCR master mix. The vials intended for the HPV negative and positive PCR controls remain closed. Cap each vial after the addition of DNA before proceeding with the next.
8. Mix HPV positive PCR control (vial B1) on a Vortex mixer, add 10 μL to the appropriate vial, and close the vial.
9. Use clean filter tips for each sample and mix each sample with the PCR mix by pipetting up and down a few times.
10. Insert the vials into the automatic thermal cycler and run the appropriate cycling program:
  9 min at 94 °C
  40 cycles of:
    30 s denaturation at 94 °C
    45 s annealing at 52 °C
    45 s elongation at 72 °C
  3 min at 72 °C (final elongation)
  Keep the amplified products at 2–8 °C for short-term storage or freeze at −20 °C for long term storage.
11. In the second step, the amplified fragments undergo a line probe assay by reverse-hybridization assay performed by using the portion of the kit named RHA kit HPV SPF10-LiPA25. In this second step, the biotinylated amplicons are denatured and then hybridized with specific oligonucleotide probes immobilized as parallel lines in membrane strips. After immobilization of the oligonucleotides on the strips, these were washed, and streptavidin alkaline phosphatase (SAP) is added to bind to the biotinylated hybrid formed, yielding a purple precipitate in the strip that visually determines the specific HPV type compared to the kit-provided controls. The LiPA strips are visually inspected and interpreted following the standardized reference guide provided by the kit’s manual.
12. Prewarm the vials with the hybridization Solution (HS) and the Stringent Wash Solution (SW) to at least 37 °C but must not exceed the hybridization temperature of 49 °C (all crystals should be dissolved before the opening of the vial).
13. Use 2 mL of hybridization solution for each strip + 15 mL in excess for each run.
14. Use 6 mL of stringent wash solution for each strip + 30 mL in excess for each run.
15. Rinse solution (RS) should be prepared by diluting the concentrated rinse solution 1/5 (1 part concentrated solution + 4 parts of water). Prepare 10 mL rinse solution for each strip + 55 mL in excess.
16. Conjugate (C) solution and substrate solution should be prepared by diluting the concentrated conjugate or substrate 1/100 in conjugate diluent or substrate buffer, respectively. Mix gently.
17. Prepare 2 mL conjugate solution and substrate solution for each strip + 5 mL in excess for 22 or less strips.
18. In order to reduce the excess of buffer for each run with 22 or less strips, use a 50 mL conical tube and place it in the bottle provided with the ProfiBlotTM 48 T. When testing more than 22 strips an excess of 20 mL for each run is needed.
19. Use 2 mL of substrate buffer for each strip + 5 mL in excess for each run with 22 or less strips. In order to reduce the excess of buffer for each run with 22 or less strips, use a 50 mL conical tube and place it in the bottle provided with the ProfiBlotTM 48 T.
20. When testing more than 22 strips an excess of 20 mL for each run is needed.
21. All vessels used to prepare conjugate and substrate solutions should be cleaned thoroughly and rinsed with water.
22. The next step is a stringent wash using rinse solution and conjugate.
23. Remove the tray from the water bath
24. Aspirate the liquid with a pipette. Add 2ml prewarmed SW to each strip.
25. Shake the strips for 10 to 20 s at room temperature (RT). Aspirate the solution.
26. Repeat the washing step.
27. Aspirate the solution.
28. Add 2ml of SW to each strip.
29. Incubate in the shaking water at 50 °C for 30 min. Close the lid of the bath.
30. Aspirate the Stringent Wash Solution.
31. The final step is the strip color development. These steps will be at RT on a shaker (not on a water bath).
32. Wash each strip twice for 1 min using 2 mL of Rinse Solution (RS). Aspirate.
33. Add 2 mL of C (conjugate solution) to each sample and incubate for 30 min while shaking. At this point, prepare the substrate solution (S).
34. Aspirate the C solution.
35. Wash each strip twice for 1 min using 2ml of RS. Aspirate.
36. Wash once more using 2 mL of substrate buffer (SB). Aspirate.
37. Add 2 mL of the S solution an incubate for 30 min in the dark (cover with aluminium foil) while shaking. Stop the colour development by washing the strip twice in 2 mL of water while shaking for 3 min.
38. Remove and place the strip with forceps in absorbent paper.
39. Wait for the strip to dry completely.
40. Compare strip bands with the interpretation sheet provided with the kit.

#### 2.2.2. 16S rDNA (Bacterial) Read Quality Control and Data Analyses

1. Currently, it is cheaper to extract good quality DNA and send directly to any company for outsourced sequencing of 16S rDNA genes.
2. To characterize the microbiota, one sequences the V4 hypervariable region of the 16S ribosomal RNA using the universal bacterial primers: 515F (5′ GTGCCAGCMGCCGCGGTAA 3′) and 806R (5′ GGACTACHVGGGTWTCTAAT 3′) as described in the Earth Microbiome Project (EMP; http://www.earthmicrobiome.org/emp-standard-protocols/16s/;) [27].
3. Once the facility sends the data, raw FASTQ reads can be uploaded to QIITA https://qiita.ucsd.edu/, an entirely open-source microbial study management platform [28]. It allows users to keep track of multiple studies with multiple omics data, and use stringent quality criteria PHRED scores (quality score of the nucleotides generated by automated DNA sequencing) with an ASCII offset of 33, and the user can choose the length of the reads for trimming, demultiplexing and binning using a given database (Greengenes, SILVA, or the Ribosomal Database Project-RDP). The usefulness of this platform is that it allows data to be directly made public via EBI (the European Bioinformatics Institute), a great service offered by the QIITA team at the University of California San Diego (UCSD).
4. After the OTU table is prepared in QIITA, an alternative is to use QIIME2 for analyses or R packages such as Bioconductor [29] or the quick tool microbiomeanalyst (https://www.microbiomeanalyst.ca) for preliminary analyses [30].
5. Reads matching chloroplast, mitochondria, and unassigned sequences should be removed as well as rare singletons.
6. Downstream taxonomy plots, alpha and beta analyses should follow a given rarefaction level considering a minimum number of reads shared by most samples.
7. Taxonomic barplots, alpha richness (number of OTUs/Operational Taxonomic Units) and geometric diversity boxplots (Shannon index of equitability [31]), can be built using R’s ggplot package [32].
8. Heatmaps can be built using the plot_heatmap function from the heatmap library [33].
9. Additional analyses can be plotted using a microbiomeanalyst that integrates multiple R programs [30] and also includes LEfSe (Linear discriminant analysis effect size) analyses [34].
10. For beta diversity, samples can be visualized using pairwise Bray–Curtis distance between samples using the R package [35] with vegdist function in vegan [36]. The global differences in bacteria can be visualized with Principal Coordinates Analysis (PCoA) or non-metric multidimensional scaling (NMDS). Alternatively, beta diversity can be visualized using UniFRAC [37].

### 2.3. Collection of Urine Samples for Metabolomics

All reagents must be an analytical grade (HPLC). All aqueous solutions used throughout this protocol should be prepared with Milli-Q or deionized water (18.2 MΩ-cm, at 4°). 

When possible, all procedures should be performed using glass appliances. The whole extraction needs to be performed at 4 °C since the metabolites can degrade as little as a few seconds.

1. Wet ice
2. Gloves
3. Sterile 4oz Specimen Cups
4. Eppendorf vials of 1.5 mL
5. Zip-Lock bags

### 2.4. Extraction of Metabolites from Urine

1. An amount of 200 μL of liquefied urine samples
2. Extraction solution: Methanol/Water mixture 8:1 (by volume)
3. Borosilicate glass disposable culture tubes 16 × 100 mm
4. Glass pipettes, 10 mL
5. plastic beaker, 50 mL
6. Pasteur pipettes and pipette rubber bulbs
7. Vortex
8. Reaction tube centrifuge (e.g., Eppendorf 5810 R)
9. Vials, screw top, clear glass, 1.5 mL, thread 8-425
10. Assembled screw cap with the hole with PTFE/silicone septum, thread 8-425 (Cat# 27093-U Sigma-Aldrich)
11. Plastic caps solid-top, thread 8-425
12. Rotary vacuum evaporator

### 2.5. Metabolites Derivatization

1. Derivatization solution 1: 20 mg/mL Methoxyamine hydrochloride in pyridine (see Note 1)
2. Derivatization solution 2: *N*-*tert*-Butyldimethylsilyl-*N*-methyltrifluoroacetamide with 1% *tert*-Butyldimethylchlorosilane (MTBSTFA+1% TBDMSCl, mixture 99:1, Cat # 375934-10X1ML, Sigma-Aldrich)
3. Glass pipettes, 10 mL
4. Vials, screw top, clear glass, 1.5 mL, thread 8-425
5. Black plastic caps with a solid top, white rubber liner, thread 8-425
6. Vials, screw top, clear glass, 4 mL, thread 13-425
7. Black plastic caps, solid top, white rubber liner, thread 13-425
8. Dry Block Incubator for glass vials (diameter of the well ~14 mm)
9. Eppendorf vial of 1.5 mL
10. Reaction tube centrifuge (e.g., Eppendorf 5415D)
11. Glass inserts (Cat # 29445-U, Sigma-Aldrich) (see Note 2).
12. Assembled screw cap with a hole with PTFE/silicone septum, thread 8-425 (Cat# 27093-U Sigma-Aldrich
13. Hexane ≥ 95% for HPLC

#### 2.5.1. Gas Chromatography/Mass Spectrometry (GC/MS) Analysis and Data Acquisition

1. GC/MS
2. Fused-silica capillary column RXI-5MS (0.25 mm inner diameter, 0.25 μm D.F., 30 m)
3. Hexane ≥ 95% for HPLC
4. NIST/EPA/NIH Mass spectral Library (NIST14)

#### 2.5.2. Extraction of Metabolites

1. Separate 200 μL of liquefied urine samples and place them on ice.
2. Mix with 800 μL of the cold methanol-water mixture (8:1 *v*/*v*)
3. Vortex mixture for 1 min.
4. Keep the sample at 4 °C or ice for 20 min.
5. Centrifuge at 6000 rpm for 8 min at 4 °C.
6. De-assemble the screw cap and remove silicone septum. Perforate it using scissors and mount vials onto a rotary vacuum evaporator. (see Note 3).
7. Remove 200 μL of the supernatant from the top phase and transfer to a glass vials, thread 8-425.
8. Prepare reference pool quality control samples (see Note 4).
9. Attach vials to a rotary vacuum evaporator (see Note 5).
10. Evaporate supernatants to dryness.
11. Replace caps with solid tops.
12. Put vials in desiccator and store at −80 °C for at least four weeks.

#### 2.5.3. Metabolites Derivatization

1. Prepare the derivatization solution 1: mix 20 mg Methoxyamine hydrochloride in 1 mL pyridine using 4 mL glass vials with plastic caps solid-top, thread 13-425 (see Note 6). Briefly, vortex the obtained solution at RT until Methoxyamine hydrochloride is fully dissolved.
2. Take out dried samples from storage and allow them to warm up to room temperature for at least 15 min before derivatization (see Note 7) and add 50 µL Derivatization Solution 1, close tightly the vials with closed caps and incubate at 37 °C for two hours.
3. Remove samples, add 50 µL of the Derivatization Solution 2 (see Note 8) directly to the reaction mixture in the vial, close tightly the vials with closed caps and incubate for an additional 1 h at 60 °C.
4. Transfer the reaction mixture to labeled Eppendorf vial and centrifuge at 13,000 rpm for 10 min at RT.
5. Transfer the supernatant in the new glass vial and immediately close each sample with a solid plastic cap, thread 8-425. Store for a long time at −20 °C and for a short time at 4 °C (see Note 9).
6. For the analysis, place a glass insert in the new glass vial and dilute each sample 1:50 with hexane prior to GC/MS analysis (see Note 10).

### 2.6. GC/MS Analysis

1. The mass spectrometer must be tuned according to the manufacturer’s manuals for optimal parameters for ion lenses, detector voltage, and other settings. Usually, this can be performed in autotune operation.
2. Inject 1 μL of each sample including quality control samples in the GC-MS.
3. Separate metabolites using a GC temperature ramping program. The GC oven can be programmed from 100 °C to 280 °C at a rate of 4 °C/min. The injection port temperature will be 280 °C, and helium is used as the carrier gas at a constant linear velocity of 39 cm/s. Injection mode—split (15:1).
4. Detect metabolites by setting the ion source filament energy to 70 eV, the ion source temperature −200 °C, and the scan range (mass-to-charge, *m*/*z*) 35–600 Da.

#### Metabolomic Data Acquisition and Analyses

**Figure 2:**
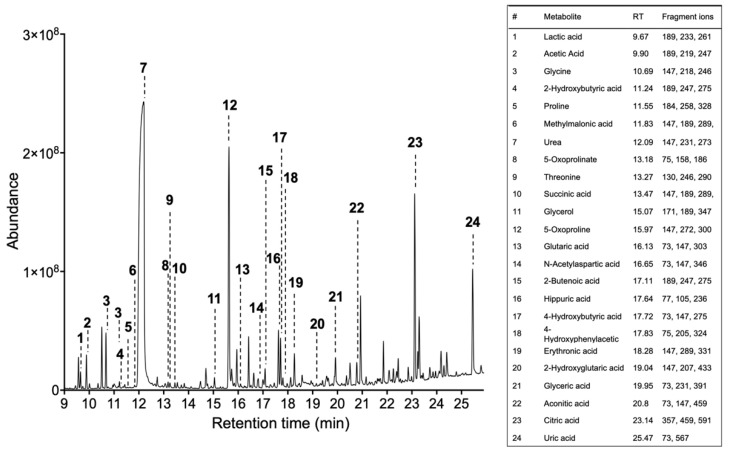
GC-MS total ion current chromatogram (TIC) of metabolites extracted from human urine samples. RT—retention time, ID—metabolite identity, CFI—characteristic fragment ions, %—identification probability value.

1. Obtain the total ion chromatogram and mass chromatograms for each detected metabolite GC/MS instruments generate a single file per sample, a list of mass spectra together with their corresponding retention times (RT). These spectra are commonly shown on a chromatogram represented by RT on the horizontal axis and signal intensity on the vertical axis (Figure 2).
2. Identify metabolites based on the fragmentation pattern of molecular ions (characteristic fragment ions) using the GC/MS manufacturer’s software equipped with NIST/EPA/NIH Mass spectral Library (NIST14) (see Note 11).

### 2.7. Statistical Analysis 

#### 2.7.1. Microbiota

1. Statistical tests on the beta diversity can be completed via community-level differences between sample groups, assessed using the PERMANOVA test, which allows sample-sample distance to be applied to an Analyses of Variance (ANOVA)-like framework [38]. This PERMANOVA test is determined through permutations and provides strength and statistical significance on sample groupings using a Bray–Curtis distance matrix as the primary input.
2. Analyses of Variance tests using the aov function in R (Team, 2008) must be used on the rarefied richness (alpha) values as well as for the Shannon diversity values, to find significant differences related to a given category. *p*-Values should be considered using an FDR of 0.05 to be considered significant.
3. The differential abundance test for OTUs, to identify possible signatures significantly associated with the metadata categories (biomarkers), can be completed with non-parametric Wilcoxon rank sum tests that have lower false positive rates and are more robust to outliers, without needing a normal distribution assumption.
4. Biomarker analyses can be identified also with LEFSE [34].

#### 2.7.2. Metabolomics

1. Quantify each peak using the maximum peak intensity value and organize data as a “peak intensity table” using MS Excel or similar software (see Note 12). The table must include metabolite identities and peak intensity values and saved in Comma Separated Values (.csv) or Tab Delimited Text (.txt). Perform statistical analyses using free online tool MetaboAnalyst.ca [39,40] (see Note 13).
2. Open MetaboAnalyst.ca, choose “Statistical Analysis”, upload the “peak intensity table” and submit. If the table was organized correctly and saved in the required format, the “Data Integrity Check” will pass the information for further analysis. Click the “Skip” button to accept the default practice (see Note 14).
3. In “Data Filtering” click “None”.
4. Perform data normalization to remove unwanted variations between the samples. First, perform “sample-specific normalization. Click “Proceed” (see Note 15).
5. For two groups (control and experimental) perform univariate analysis (fold change analysis, T-tests, volcano plot, correlation analysis).
6. For more groups, perform chemometrics analysis (Principal Component Analysis (PCA), Partial Least Squares-Discriminant Analysis (PLS-DA), feature identification and cluster analysis. Detect outliers to improve downstream results (see Note 16).

## 3. Multi-Omic Integration of Microbiota and Metabolomics

The integration of metagenomics and metabolomics data could be performed using Model-based Integration of Metabolite Observations and Species Abundances 2 (MIMOSA2) freely available at borensteinlab.com/software_MIMOSA2.html [41,42]. MIMOSA2 summarizes paired microbiome–metabolome datasets to support mechanistic interpretation and hypothesis generation. MIMOSA2 also characterizes the relative capacity of community members to produce or consume metabolites based on a priori metabolic information of the activity of metabolic enzymes for each species from the KEGG database, describes how well each metabolite can be predicted by metabolic potential, and estimates how much each taxon can explain each metabolite.

1. Prepare the output data files and save in txt format for each sample in either control or experimental groups separately.
  - For “microbiome”: From QIITA or QIIME, export the species table (BIOM) with Greengenes ID as OTU identifiers [43]. This will allow for PICRUST [44] to use the OTU table and result in a functional-gene-count matrix, telling the count of each functional-gene in each of the samples surveyed, based on the reference genome.
2. For “metabolome”—use KEGG [45] IDs for each identified metabolite and it’s identified concentration.
3. Log in to borensteinlab.com/software_MIMOSA2.html. The following options will be used for the analysis:
  B Microbiome data—create a table using Greengenes 13_5 or 13_8 OTUs;
  C Metabolic model settings—PICRUSt and KEGG metabolic model;
  D Metabolome data—create a table using KEGG compound IDs, that can be logarithmically transformed if required;
  E Algorithm settings—the least-squares (OLS) regression estimation could be used for comparing metabolite levels with community metabolic potential (CMP) scores identified with positive model slope and a model *p*-value < 0.1. (see Note 17).
  F Log in to borensteinlab.com/software_MIMOSA2.html.
4. MIMOSA2 will calculate CMP scores for each metabolite and generate a tab-delimited file for each bacterial species in each condition. Additionally, a contribution table will be generated as a tab-delimited file which will relate taxa, to KEGG metabolic pathways with its associated *p*-value, model slope and VarShare (represent the fraction of the variation) for each metabolite explained by the taxon in question. VarShare values could be used to build heatmaps in R, in the same format as those for microbial taxa. Finally, MIMOSA2 will identify microbial features that may underlie differences in microbial metabolite concentrations between similar communities. It answers questions such as: (1) Do the metabolites in a given dataset appear to vary depending on microbiome composition? Which ones? (2) Can differences in microbiome metabolic capabilities explain metabolite variation? (3) Which taxa, genes, and reactions appear to be playing a role in metabolite differences? [42].

### Notes

1. Methoxyamine hydrochloride is very sensitive to moisture. Storage is recommended under the vacuum at RT.
2. Glass inserts decrease the surface area inside the vial allowing to achieve maximum sample recovery and easier sample removal.
3. De-assemble the screw cap and remove silicone septum. Perforate it using scissors and mount the vials onto a rotary vacuum evaporator. This step is required to minimize sample cross-contamination during evaporation.
4. Preparation of the reference pool quality control samples. Prepare a large pool sample during the preparation of sample extracts by aliquoting 100 µl from each 1 mL authentic subject sample. After a collection of a large pool sample, aliquot all for 1 mL in separate vials (thread 8-425), label all as reference quality controls and process similar to authentic subject samples. Continue as instructed in Section 3.3.7. The derivatization solution without the sample only, and empty vials, must also be included as quality controls in the analysis to ensure that identified metabolites are derived from the sample [46].
5. Attach the glass vials to the rotary evaporator [47].
6. Derivatization Solution 1 must be freshly prepared.
7. During derivatization, avoid contact with moisture, which will result in metabolite degradation. If samples are not dried, it is necessary to proceed to additional evaporation as said in Sections 3.4.9–3.4.11.
8. Derivatization Solution 2 must be used from freshly opened vials.
9. If an analysis is performed after samples preparation, wait two hours before injecting the first sample into the GC-MS.
10. The sample concentration must be adequately adjusted to give sufficient chromatogram, then it needs to be concentrated before injecting the sample into the gas chromatograph. The dilution of a sample (1:50) was found to be sufficient for the analysis. However, if the signal is too low, the concentration could be increased. Derivatization solution without the sample only, and empty vials, must also be included as quality controls in the analysis to ensure that identified metabolites
11. MS detects the mass of the molecular ions and the masses of the fragment ions. The database contains extensive information on many compounds including the masses of fragment ions that help to perform successful metabolite identification. In addition, to ensure a valid peak identification, data can be also processed using a deconvolution method, which is a signal processing technique that estimates the relative area corresponding to each peak when multiple peaks overlap within the same spectral region. This is especially important for low abundant metabolites that might co-elute with abundant major peaks. For general quadrupole mass spectrometers, data deconvolution by the freely available software AMDIS is recommended (http://chemdata.nist.gov/mass-spc/amdis/).
12. Some of the metabolite’s so-called “missing values” will not be identified in chromatograms. Therefore, when composing the table for the analysis, missing values should be presented as empty values or NA without quotes.
13. MetaboAnalyst is a web server designed to permit comprehensive metabolomics data analysis, visualization, and interpretation [39]. The web interfaces of MetaboAnalyst are designed to be self-explanatory. Therefore, most steps are documented on top of the corresponding pages. Available tutorials and sample data sets complement the information by providing step-by-step instructions for several most common tasks.
14. This step is strongly recommended for metabolomics datasets with a large number of variables (more than 2000 metabolites) since many of them are from baseline noises. Usually, the GC/MS analysis of brain tissues could identify less than 60 variables.
15. The normalization procedures are grouped into three categories. The sample normalization allows general-purpose adjustment for differences among your sample, which can include the protein content in the sample or intensity value of internal standard spiked into the derivatization mixture before GC/MS analysis. Data can be further transformed by “log” or “cube root” and/or scaled via “mean centering, autoscaling, Pareto scaling, and range scaling” (please see the description of each in the MetaboAnalyst program). According to [48] autoscaling and range scaling performed better than the other pretreatment methods and showed biologically sensible results in chemometrics analysis. Data transformation and scaling are two different approaches to make individual features more comparable that can be used individually or in combination. Scaling is a useful procedure when variables are of very different orders of magnitude [49].
16. Detect outliers. This can be carried out by visual inspection of the PCA plot to identify samples-outliers outside the Hotelling T2 95% confidence ellipse [40]. Many outliers could be corrected by normalization or excluded from the analysis. In many cases, outliers are the result of operational errors during the analytical process. If those values cannot be corrected, they should be removed from the analysis, but always justified [39]. Please refer to MetaboAnalyst.ca tutorials, or FAQ to select the statistical analysis method.
17. Well-predicted metabolites were identified by the Community Metabolic Potential (CMP) scores model in groups by examining of the total pool of metabolites with positive model slope and a model *p*-value < 0.1. Given that MIMOSA2 is prone to false negatives because its model is approximate, it requires a weak statistical threshold (*p*-value < 0.1) to capture more relationships of possible interest [18,41,42]. Additional information could be found at borensteinlab.com/software_MIMOSA2.html helper.

## 4. Discussion and Conclusions

We are assisting a new stage of microbiome research, which moves beyond the typical 16S profiles listing bacteria associated to a given body site or disease, and offers the novel integration of other omics approaches, towards a better understanding of community functions in the disease, and interactions with the host. Emphasis is now given to the causality of microbiome research and the development of mechanistic understandings of the influence of microbiota, including their metabolic activities. Thus, the integration of metagenomics and metabolomics is becoming a very promising approach in the exploration of associations between the host microbiome and circulating metabolites with the aim of understanding the complex bacterial interactions with the host and their contributions to systemic health. Using urine as a liquid biopsy is gaining more popularity. Indeed, evidence suggests that biomarkers for cervical cancer may be exfoliated as debris in urine, which could facilitate diagnostics [50]. Since circulating metabolites are finally excreted through urine [51], recent studies suggest that exploring the association between urine metabolome and host microbiome (gut or even cervicovaginal) may complement sequencing-based approaches with a functional readout of the host microbiome and its interaction with host [52,53]. Indeed, a recent study coupling gut microbiome and urine metabolomics suggests that gut bacterial compositional changes could eventually be monitored and probed using urine metabolomics [52]. In addition, the application of the current protocol for functional characterization of vaginal and cervical hr HPV microbiota and urinary metabolome has identified species-contributors to the pool of several circulating urinary metabolites associated with cervical cancer development [18]. The high throughput analyses of these two datasets could be key for the development of new tools (like portable devices) to give women feasible self-testing, and to allow them to be informed of their cervicovaginal dysbiosis via vaginal or urine metabolomic screening, which could benefit low-income countries, and those with a lack of access to cervical surveillance.

Taken together, this report is a simple and easy-to follow protocol, aimed to be used as a guide for those taking on microbiota and metabolic analyses using human biospecimens, such as cervicovaginal microbiota and urine metabolomics [5,18,52]. Data integration via the use of freely available MIMOSA2 software [42] facilitates analyses providing easy export files that can be plotted in any other widely used packages such as R [54]. Generating data that combines both the microbiome and metabolomics analyses will guide the development of tools aimed at detecting dysbiosis via changes of the microbial profiles and metabolomic signatures, which will be useful for new point-of-care diagnostics.
